# Simulation enhanced distributed lag models for mortality displacement

**DOI:** 10.1186/s40064-016-3566-6

**Published:** 2016-11-10

**Authors:** Koen Simons, Ronald Buyl, An Van Nieuwenhuyse, Danny Coomans

**Affiliations:** 1 Health and Environment, Department of Food, Medicine and Consumer Safety, Scientific Institute of Public Health, Juliette Wytsmanstraat 14, 1050 Brussels, Belgium; 2 Department of Biostatistics and Medical Informatics, Public Health, Vrije universiteit Brussel, Laarbeeklaan 103, 1090 Jette, Belgium

**Keywords:** Air pollution, Distributed lag model, Harvesting, Mortality displacement, Simulation study, Time series

## Abstract

Distributed lag models (DLM) are attractive methods for dealing with mortality displacement, however their estimates can have substantial bias when the data is generated by a multi-state model. In particular DLMs are not valid for mortality displacement. Alternative methods are scarce and lack feasibility and validation. We investigate the breakdown of DLM in three state models by means of simulation and propose simulation enhanced distributed lag models (SEDLM) to overcome the defects. The new method provides simultaneous estimates of the net effect (entry) and the displacement effect (exit). These have improved performance over the singular estimate from a regular DLM. SEDLM entry estimates have negligible bias and their variance is reduced. The exit estimates are unbiased and their variance is one order of magnitude lower with respect to the entry estimates. Applying SEDLM to the original Chicago data, the 95% highest posterior density intervals for both entry and exit contain 0, providing neither evidence for a ‘displacement effect’ nor for a ‘net effect’.

**Electronic supplementary material:**

The online version of this article (doi:10.1186/s40064-016-3566-6) contains supplementary material, which is available to authorized users.

## Background

Distributed lag models (DLM) have become the dominant approach for modelling acute mortality effects of environmental exposures such as atmospheric ozone (Zanobetti and Schwartz 2008), fine particulate matter (Zanobetti et al. 2002), ambient temperature (Basu 2009), heat waves (Hajat et al. 2002) etc.

Several arguments favour DLMs. Primarily, they provide an intuitive way of estimating risks when the delay between exposure and event is unknown or variable. Secondly, DLMs are flexible and have been extended to investigate thresholds (Muggeo 2008) and non-linear exposure-response relationships (Gasparrini et al. 2010). Interactions between exposures have also been included (Filleul et al. 2006, e.g.). Thirdly, DLMs are fairly easy to implement in standard statistical software. And lastly, DLMs were considered to give both quantitative and qualitative information on mortality displacement.

**Fig. 1:**
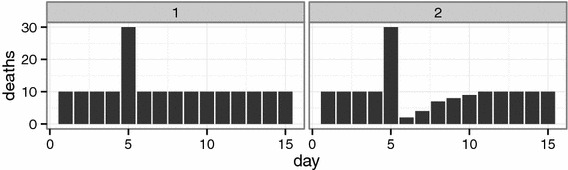
Two scenario’s for daily mortality influenced by a peak in air pollution exposure on day five. *Left* twenty additional deaths, no displacement. *Right* The increase in mortality is followed by a lowered mortality and the total deaths over fifteen days is equal to what would be expected without a peak in pollution. The twenty deaths are ‘displaced’ by only a few days

Under the mortality displacement hypothesis not all attributable deaths originate in the general population, but weakened, near-death individuals are ‘targeted’ first by environmental exposures such as air pollution. As this ‘frail population’ depletes, mortality rates can drop below the baseline as is illustrated in Fig. 1. Vice versa, when fitted relative risks are significantly below the value of one for one or more lags, this is considered evidence of mortality displacement.

The distinction between weakened individuals and the general population is important for impact calculations. The general population enjoys a residual life expectancy of several years or decades. Consequently, when air pollution episodes increase mortality rates in this group, the impact on life expectancy is large. However, in the frail group residual life expectancies are very short. Hence, an exposure event can bring deaths in the frail group forward only some days or weeks and the effect on life expectancy is negligible in comparison with the general population.

Naturally, it is possible that there is an effect on both groups. In such a scenario it is preferable to disentangle the effects and consider only the effect on the general population when calculating the impact on life expectancy. Often the sum of the lag estimates generated by a DLM, is used, as the individual lag estimates can be both positive and negative. In essence both the left and right pattern of Fig. 1 are allowed for by a DLM.

Unfortunately, it was shown in Roberts and Switzer (2004) that such estimates are biased. This bias does not seem to be an attenuation nor a consistent over-estimation. Instead their results show that the bias depends in some non-trivial manner on the number of lags included in the model, on the size of the true effect and on the mean lifetime in the frail population. Although the bias was negligible in some scenario’s, it was sufficiently large to lead to spurious conclusions in other settings.

Nonetheless, DLMs remain the most popular class of models for acute effects of air pollution exposure. We believe that two factors contribute to this. Firstly, the motivation for the DLM is intuitive and although it has, to our knowledge, never been proven analytically that the estimates should be unbiased, it is difficult to understand why they are sometimes biased towards the null, yet at other times unbiased or biased away from the null. Secondly, there is no well-established alternative model. Even though alternative models have been proposed as early as 1999 (Smith et al. 1999), they have not been extensively studied. In particular, the aforementioned, Bayesian, approach suffered a large computational burden and only eighteen simulations were performed, all in the setting of zero mortality displacement. Other methods similarly lack feasibility or validation.

In this paper we revisit the multi state framework necessary for models that generate mortality displacement. Thereafter we recapitulate the DLM approach and provide additional observations pertaining the factors that contribute to the bias of DLM estimates. Using these insights as heuristics, we propose an intuitive modification of the DLM that corrects for bias: Simulation Enhanced Distributed Lag Models (SEDLM). Subsequently, we compare SEDLM to DLM with a simulation study and demonstrate that the SEDLM produces better estimates. We apply the method to the Chicago data as an illustrative example. Finally, we turn to the discussion and conclusion.

## Mortality displacement

**Fig. 2:**
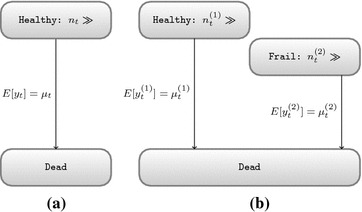
Two multi state models that do not generate mortality displacement. **a** Basic model. **b** Multiple initial states

In order to generate mortality displacement, a multi state model with at least three states is needed. However, we will first consider the simple model depicted in Fig. 2a. Herein there are only two states: healthy and dead. All people start in the healthy state and there is only one possible transition: towards death. Let us assume that the base rate of dying is sufficiently low and the population large enough that the amount of healthy people ($n_t$) is approximately constant. Thus daily mortality can be considered a Poisson process with rate $\mu _t$ that is influenced by concurrent and recent exposure including lags of up to *L* days: $x_t$, $x_{t-1} \ldots x_{t-L}$. Let us assume for simplicity that the effect is linear: ${\mathrm {log}}\mu _t = \alpha + \beta _0 x_t + \cdots + \beta _L x_{t-L}$. Unless the exposure has a protective effect, none of the $\beta _i$ can be negative. If we believe that model in Fig. 2a is the underlying truth, then we should restrict our estimates to be non-negative ($\hat{\beta _i} \ge 0$).

Next, we consider models with multiple starting groups such as shown in Fig. 2b. Herein we allow the initial states to differ in size. From each initial state there must be a direct path towards the dead state, however exchanges between groups are not allowed. Under similar conditions as for the two state model, the population sizes $n_t^{\left( 1\right) }$ and $n_t^{\left( 2\right) }$ are considered constant. Consequently, the total number of daily deaths follows a Poisson distribution with expected deaths equal to the sum of expected deaths in both groups and the overall relative risk is a weighted sum of the relative risks per population, which cannot be negative unless the exposure has a protective effects on at least one population.

**Fig. 3:**
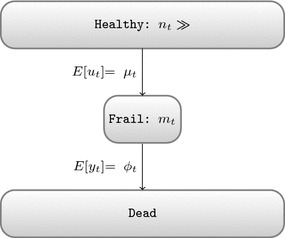
A minimal multi state model that can generate mortality displacement

In contrast, the model described in Fig. 3 does allow reduced risks and hence mortality displacement. Like the previous model, it contains three states, but there is only one initial state. All people start in the healthy state and can move towards the frail state; only those in the frail state may die. Again we assume that the healthy state has a near infinite population ($n_t$), however the frail state contains only a limited amount of people ($m_t$) and is dynamic:

1 $$ {\left\{ \begin{array}{ll} n_t \sim c \\ m_{t+1} = m_{t} + u_{t} - y_{t} \end{array}\right. } $$

On day *t*, $u_t$ healthy people become frail. We will refer to this transition from healthy to frail as entry. Similarly, on day *t*, $y_t$ frail people die and we will refer to this transition as exit. Assuming that the healthy population is large and that the individual probability of entry is small, we assign a Poisson distribution to $U_t$. In addition, we assume that initially there is only a small finite number of frail individuals and that the probability of exit is high for all individuals in the frail state. This can be modelled with a Binomial distribution. As a direct consequence of these choices, the amount of people in the frail state varies from day to day. While the outflux is proportional to the size of the frail population, the influx is independent thereof. It is easy to see that the size of the frail state will evolve towards a stable equilibrium ($E \left[ m_t \right] = E \left[ m_{t-1} \right]$) wherein the expected influx is equal to the expected number of deaths. If the outflux is too large, then the frail population will shrink and hence the outflux will decrease towards the equilibrium state. Vice versa, a too small frail population will experience a net increase until its size, multiplied by the base rate, becomes equal to the expected influx.

Coupled with a peak in exposure, this mechanism allows mortality displacement. If only the exit rate is increased by an exposure event, then it will immediately lead to an increase in mortality that is not compensated for by extra people becoming frail. On subsequent days fewer people risk death until the size of the frail population has recovered. This creates a pattern similar to the right panel of Fig. 1. In a more general model, pollution can alter both the rate of becoming frail and the risk of death for a frail individual:

2 $$\begin{aligned} {\left\{ \begin{array}{ll} m_{t+1}  = m_{t} + u_{t} - y_{t} \\ u_t  \sim {\mathrm {Poisson}} \left( \mu _t\right) \\ y_t  \sim {\mathrm {Binomial}} \left( \phi _t, m_t\right) \\ {\mathrm {log}} \left( \mu _t\right)  = \alpha _{{\mathrm {entry}}} + \beta _{{\mathrm {entry}}} X_t + s_1\left( t\right) + s_2\left( {\mathrm {covariates}}_t\right) \\ {\mathrm {logit}} \left( \phi _t\right)  = \alpha _{{\mathrm {exit}}} + \beta _{{\mathrm {exit}}} X_t \end{array}\right. } \end{aligned}$$

wherein $X_t$ is the concurrent exposure and $s_1\left( t\right)$ and $s_2\left( {\mathrm {covariates}}_t\right)$ are smooth functions of time and time dependent covariates. These are included in the entry rate ($\mu _t$) to allow seasonal effects in observed mortality because at equilibrium the expected daily deaths are equal to the expected daily amount of people becoming frail. On the contrary, low frequency variations in the probability of exit ($\phi _t$) do not alter the seasonal pattern of daily mortality, but are reflected in (inverted) changes in the size of the frail population.

## DLMs

If both the series of daily deaths $y_t$ and the daily population at risk $m_t$ are observed, estimates of $\beta _{{\mathrm {entry}}}$ and $\beta _{{\mathrm {exit}}}$ can be obtained with Generalized Additive Models (Hastie and Tibshirani 1990) using only concurrent exposure. However, in environmental epidemiology $m_t$ is usually not observed. Instead, a reduced model is fit on the observed data:

3 $$ {\left\{ \begin{array}{l} y_t \sim {\mathrm {Poisson}} \left( \nu _t\right) \\ {\mathrm {log}} \left( \nu _t\right) = \alpha + \beta _0 X_t + s_1\left( t\right) + s_2\left( {\mathrm {covariates}}_t\right) \end{array}\right. } $$

If $\phi _t \equiv 1$, death in the frail state is immediate and model 2 reduces to model 3. However, if $\phi _t \equiv c_2 < 1$, the reduced model is likely to underestimate $\beta _{\mathrm {entry}}$ as the excess deaths are spread out over the days or weeks subsequent to the exposure. An intuitive solution is to add *L* lagged exposure terms to capture the full effect of exposure:

4 $$ {\left\{ \begin{array}{ll} y_t  \sim {\mathrm {Poisson}} \left( \nu _t\right) \\ {\mathrm {log}} \left( \nu _t\right)   = \alpha + \beta _0 X_t + \beta _1 X_{t-1} + \cdots + \beta _L X_{t-L} + s_1\left( t\right) + s_2\left( {\mathrm {covariates}}_t\right) \end{array}\right. } $$

thus the effect of an event is distributed over multiple lags. The sum of the lagged effect estimates is often used as a measure for the total impact and it is assumed that this effect is equal to the true entry effect:

5 $$\begin{aligned} E\left[ \sum \limits _{i=0}^{L} \hat{\beta _{i}}\right] \approx \beta _{{\mathrm {entry}}} \end{aligned}$$

When $\phi _t \equiv 1$, this assumption is valid and the approximation is exact even when $L=0$. However if the base probability of dying in the frail state decreases, the mean lifetime increases and a larger number of lag terms must be included for the assumption to be plausible. As *L* increases so does the amount of degrees of freedom used. Furthermore the lagged exposure terms are anything but orthogonal. The combined effect is a substantial increase in the variance of the estimates. Adding conditions for the coefficients can reduce the number of degrees of freedom used and thus improve the model fit. One alternative is restricting the $\beta _i$ to a low order polynomial (Almon 1965; Schwartz 2000).

6 $$\begin{aligned} {\left\{ \begin{array}{ll} {\mathrm {log}} \left( \mu _t\right) = \alpha + \sum \nolimits _{j=0}^{p} \beta ^{\star }_{j} X_{j}^{\star } + s_1\left( t\right) + s_2\left( {\mathrm {covariates}}_t\right) \\ X_{j}^{\star }\left( t\right) = \sum \nolimits _{i=0}^{L} i^j X_{t-i} \\ \beta _i  = \sum \nolimits _{j=0}^{p} \beta ^{\star }_j i^j \end{array}\right. } \end{aligned}$$

Together with assumption 5, this is a Polynomial Distributed Lag Model (PDLM) of order p, with L lags. Other options include, but are not limited to, cubic B splines and penalized splines (Zanobetti et al. 2000).

While these approaches improve efficiency, a maximum lag *L* must still be chosen and this influences the estimates. More importantly, to our knowledge, the assumption in Eq. 5 has not been proven. On the contrary, Roberts and Switzer (2004) reported substantial bias when data had been generated from a three state model similar to the one described above, and also when more complex models such as including multiple frail states were used (Roberts 2011). Results from the 2004 paper indicate that choice of the number of lags influences the bias in estimates. When the simulation assumed a pure entry model ($\phi _t \equiv c$), estimates from distributed lag models with a lag number smaller than the mean lifetime in the frail population tended to underestimation, which is consistent with current understanding of DLM’s. Yet the pattern remained unclear for more general simulation settings: ie when $\phi _t$ is time-dependent, assumption 5 may produce both over- and underestimation.

## Bias correction through simulation

**Fig. 4:**
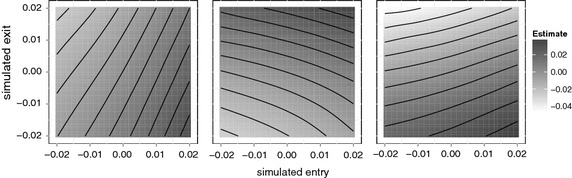
Heatmap of estimates in function of simulated entry and exit effects for a DLM with $L=40$ and B-spline restriction on the lag coefficients (*df* = 5). *Left* sum of the lag estimates. *Middle* estimate of the first component (rescaled). *Right* difference between left and middle figure. Iso-estimate lines are superimposed as a visual aid

To better understand the relationship between the parameters of the three stage model and the estimates obtained under a reduced, distributed lag model, we performed a large simulation study. The left hand side of Fig. 4 shows the estimated sum of lag effects obtained with a typical DLM. If the assumption in Eq. 5 were true, there should be a gradient in the simulated entry effect only. For the chosen DLM, this is clearly not the case. By itself, the gradient in simulated entry effect explains only 74.3% of the variation, whereas 95.6% can be explained when the simulated exit is also considered. Similar departures hold for all DLM variants we tested. In conclusion the following weaker assumption provides a much better approximation:

7 $$\begin{aligned} E\left[ \sum \limits _{i=0}^{L} \hat{\beta _{i}}\right] \approx f^{{\mathrm {model}}}\left( \beta _{{\mathrm {entry}}},\beta _{{\mathrm {exit}}}\right) \end{aligned}$$

wherein $f^{{\mathrm {model}}}$ is a smooth and model-specific function. The assumption is automatically satisfied if a similar assumption holds for all (restricted/transformed) lag estimates:

8 $$\begin{aligned} \forall j: E\left[ \hat{\beta _{j}^{\star }}\right] \approx f_j^{\mathrm {model}}\left( \beta _{\mathrm {entry}},\beta _{\mathrm {exit}}\right) \end{aligned}$$

The middle panel of Fig. 4 displays the estimates for the first component of a DLM with 40 lags restricted to a 5 degrees of freedom spline basis. The right hand side shows the weighted sum of the second to fifth components. The panels display different patterns, yet the functions appear equally smooth. By itself, none of the panels allows us to determine a unique pair of parameters (entry,exit) from a single estimate. However, the iso-estimate lines of different panels do intersect and this gives a unique solution. Unfortunately, the functions $f_j$ are unknown and therefore the curves determined by $f_j\left( \beta _{\mathrm {entry}}, \beta _{\mathrm {exit}} \right) = \beta_j$ are also unknown. Fortunately, it is possible to estimate these functions by way of simulation.

More generally, the ideal situation is such that the probability distributions $p \left(\hat{ \beta}_j \vert \beta _{\mathrm {entry}}, \beta _{\mathrm {exit}} \right)$ are known and the likelihood $L \left( \beta _{\mathrm {entry}}, \beta _{\mathrm {exit}} \vert {\hat{\mathbf{\beta }}} \right)$ can readily be transformed into a posterior for the entry and exit effects. In practise, we propose to approximate the likelihood by using simulation results. We refer to this approach as simulation enhanced distributed lag models (SEDLM):

0. Fit a DLM to the data and save the estimates $\hat{\varvec{\beta }}^{{\mathrm {realdata}}}$.
1. Simulation
  - Specify a three state model.
  - Simulate time-series with known $\beta _{\mathrm {entry}},\beta _{\mathrm {exit}}$.
  - Fit a DLM to each generated time-series, using the same specification as in step 0. Save the sets of $\lbrace \beta _{\mathrm {entry}},\beta _{\mathrm {exit}}, \hat{\beta }_0^{\mathrm {DLM}}, \ldots , \hat{\beta }_J^{\mathrm {DLM}} \rbrace$.
2. Estimate the response surfaces $\widehat{f_j^{\mathrm {DLM}}} \left( \beta _{\mathrm {entry}},\beta _{\mathrm {exit}} \right)$.
  - Choose a smoother *s*
  - $$\forall j \in 0\ldots J, \forall \beta _{\mathrm {entry}},\beta _{\mathrm {exit}}$$ : $$E\left[ \hat{\beta }_j^{\mathrm {DLM}} \vert \beta _{\mathrm {entry}},\beta _{\mathrm {exit}}\right] \approx s\left( \hat{\beta }_j^{\mathrm {DLM}} \vert \beta _{\mathrm {entry}},\beta _{\mathrm {exit}}\right) $$.
  - Calculate the residuals.
  - Estimate the local covariance matrix.
3. Compare the estimates from step 0 with the information obtained in step 2
  - $\forall \beta _{\mathrm {entry}},\beta _{\mathrm {exit}}$: calculate the Mahalanobis distance between $\hat{\varvec{\beta }}^{\mathrm {realdata}}$ and ${\varvec{s}} \left( \beta _{\mathrm {entry}},\beta _{\mathrm {exit}}\right)$
  - $\forall \beta _{\mathrm {entry}},\beta _{\mathrm {exit}}$: calculate an approximate likelihood $\widetilde{L} \approx L \left( \beta _{\mathrm {entry}}, \beta _{\mathrm {exit}} \vert \hat{\varvec{\beta }}^{\mathrm {realdata}} \right)$
  - Use numerical integration to normalize the approximated likelihood $\widetilde{L}$.

This procedure extends any DLM into a SEDLM that is appropriate for three state models. Because many variations are possible, we will consider each step in more detail before turning our attention to the data, simulation settings and results.

### Simulation

In order to simulate a time series from model 2, appropriate values for the parameters need to be chosen. The goal is to mimic the conditions of the real dataset. Thus it is a natural choice to use the real exposure and covariate time series and to fix $\alpha _{\mathrm {entry}}$ to the long-run average of the observed deaths, noting that in the long term the number of deaths equals the number of entry transitions. Similarly an estimate of the smooth seasonal variation and the smooth function of the covariates can be obtained from the real dataset.

Since no exact information on the mean lifetime in the frail state (MLT) is available, a value or a distribution must be chosen for it. Considering that a mean lifetime of several months implies a relatively large possible loss of life expectancy and that mortality displacement aims to distinguish between very small losses and larger losses, it seems logical to limit the maximum value of this parameter. Likewise, noting that $\phi _t \equiv 1$ is a degenerate case of the three state model, a mean lifetime of zero days seems of little added value. Nonetheless, limiting the MLT to a single value is not a realistic use-case. Therefore we decided to model the uncertainty of this parameter by sampling from a uniform distribution with range one to twenty eight days.

Two parameters can be derived from the MLT: the base probability of death $\alpha _{\mathrm {exit}}$ and the initial size of the frail population. In equilibrium, the expected number of new frail people is equal to the expected number of deaths. The latter is equal to the size of the frail population multiplied by the probability of dying. Vice versa, the product of the MLT and the mean daily deaths can be substituted for the initial size of the frail population.

This leaves only two more parameters to set: $\beta _{\mathrm {entry}}$ and $\beta _{\mathrm {exit}}$. In order to obtain good estimates of the surfaces $f_j$, it is necessary to evaluate them over a wide range of $\left( \beta _{\mathrm {entry}},\beta _{\mathrm {exit}}\right)$ pairs. A straightforward solution is using an envelope: drawing the pairs from a uniform distribution on a square or rectangle *S*. In order to facilitate computations, this is modified by dividing the square *S* into several smaller cells $G_i$ and drawing several pairs from uniform distributions on each $G_i$.

### Smooth response surface

Using the assumption that the $f_j^{\mathrm {DLM}}$ are continuous, a natural method for estimating them is a running mean smoother. By using a smoother with a limited span, this computation can be sped up. Any point ${\varvec{a}}$ within the envelope *S* must lie within exactly one cell $G_i$ and each cell $G_i$ has at most eight neighbours. Only points lying in the cell $G_i$ or in its neighbours, can have a non-zero weight. For each of these points, the Euclidean distance from ${\varvec{a}}$ to the center of the kernel (${\varvec{p}}$) is calculated. Subsequently a weight is assigned. We use the Epanechnikov kernel (Epanechnikov 1969) with span equal to the length (*h*) of the small squares $G_i$:

9 $$ {\left\{ \begin{array}{l} w \left( {\varvec{a}} \vert {\varvec{p}} \right)  = \frac{3}{4}\left( 1- \Vert \frac{{\varvec{p}} - {\varvec{a}}}{h}\Vert ^2_2 \right) _{+} \\ \hat{{\varvec{f}}}^{\mathrm {DLM}} \left( {\varvec{p}}\right)  = \sum _{\varvec{a}} w \left( {\varvec{a}} \vert {\varvec{p}} \right) \hat{{\varvec{\beta }}} \left( {\varvec{a}} \right) / \sum _{\varvec{b}} w \left( {\varvec{b}}\vert {\varvec{p}}\right) \end{array}\right. } $$

The local covariance matrix is estimated from the residuals (${\varvec{r}} \left( {\varvec{a}}\vert {\varvec{p}}\right) = \hat{{\varvec{\beta} }}\left( {\varvec{a}}\right) - \hat{{\varvec{f}}}^{\mathrm {DLM}} \left( {\varvec{p}}\right)$) weighted with the same $w \left( {\varvec{a}}\right)$. For computational efficiency, this process is repeated for each test point ${\varvec{p}}$ within the cell $G_i$, before moving to the next cell.

### Calculating the posterior probability by numerical integration

To calculate the likelihood of the observed DLM estimates for a given point ${\varvec{a}} \in S$ we assume that the variation of the DLM estimates around the response surfaces $f_j^{\mathrm {DLM}}$ stems from a multivariate normal distribution:

10 $$\begin{aligned} \hat{{\varvec{\beta }}}^{\mathrm {DLM}} \sim \mathrm {MVN} \left( {\varvec{{f}}}^{\mathrm {DLM}}\left( {\varvec{a}}\right) , {\varvec{{\Sigma }}}\left( {\varvec{a}}\right) \right) \end{aligned}$$

which we approximate by using the estimated smooth surfaces and estimated local covariance matrices obtained in step two:

11 $$\begin{aligned} \hat{{\varvec{\beta} }}^{\mathrm {DLM}} \sim \mathrm {MVN} \left( \hat{{\varvec{f}}}^{\mathrm {DLM}}\left( {\varvec{a}}\right) , \hat{{\varvec{\Sigma }}}\left( {\varvec{a}}\right) \right) \end{aligned}$$

Plugging in the estimates of the real data, from step zero, allows application of Bayes’ theorem:

12 $$\begin{aligned} d^2= & {} \left( \hat{{\varvec{\beta} }}^{\mathrm {realdata}} - \hat{{\varvec{f}}}^{\mathrm {DLM}}\left( {\varvec{a}}\right) \right) ^{t} \hat{{\varvec{\Sigma }}}^{-1}\left( {\varvec{a}}\right) \left( \hat{{\varvec{\beta} }}^{\mathrm {realdata}} - \hat{{\varvec{f}}}^{\mathrm {DLM}}\left( {\varvec{a}}\right) \right) \nonumber \\ p\left( {\varvec{a}}\right)= & {} {\left\{ \begin{array}{ll} 0 &\quad {\varvec{a}} \notin S \\ c \dfrac{e^{-d^2/2}}{2\pi \vert \Sigma \left( {\varvec{a}}\right) \vert ^{1/2}} &\quad {\varvec{a}} \in S \end{array}\right. } \nonumber \\ c= & {} \int \limits _{{\varvec{a}} \in S} p\left( {\varvec{a}}\right) d{\varvec{a}} \end{aligned}$$

Finally, the factor *c* is estimated by approximating the integration over *S* by a sum.

## Data and simulation setup

For maximal comparability, we based our simulations on the same dataset as Roberts and Switzer (2004). The data from the National Mortality, Morbidity, and Air Pollution Study (NMMAPS, Peng et al. (2004)) is freely available. It contains time series of daily deaths for multiple cities in the USA. These are stratified by cause of death (accidental, respiratory, pneumonia, ...) and age group (<65, 65–74, $\ge$75). It also contains time series of air pollutants and climatological variables for each city.

Following Roberts and Switzer, we selected the city of Chicago and the time period 1987–1994 from the NMMAPS database. We selected all daily deaths in residents of age 65 and above. We used PM10 as exposure and removed both outliers (daily PM10 concentration $>150\,\upmu\hbox{g/m}^3$) and missing values. These days were also removed from the mortality series. Both the temperature (*T*), dew-point temperature ($T_d$) and PM10 series were centralised.

We used a generalized linear model [GLM, McCullagh and Nelder (1989)] to obtain an estimate of the seasonal component of the mortality time series. More precisely, we fitted a Poisson model with linear predictor $s_1(t) +{\mathrm {DOW}}(t) + s_2(T_{d}(t)) + s_3(T(t)) + s_4(T(t-1))$ wherein the $s_i$ are natural cubic splines with respectively 7 degrees of freedom per year, 3, 6 and 6 *df*. DOW is the day of the week effect. We used the package mgcv (Wood 2011), R 3.0.2.(R Development Core Team 2011).

We chose a square *S* with corners (−0.02, −0.02) and (0.02, 0.02), corresponding to a maximum effect of 22% increase in mortality for each $10\,\upmu{\text{g}}/{\text{m}}^3$ of PM10. This is reasonably large with respect to current estimates, allowing for a large area away from the boundaries of the envelope while keeping the computational load low. We divided the square into 32 × 32 smaller squares and drew 80 triplets $\left( \beta _{\mathrm {entry}},\beta _{\mathrm {exit}},{\mathrm {MLT}}\right)$ for each small square, yielding a total number of 81,920 pairs. As noted in “Simulation” section, the MLT is sampled from a uniform distribution with range 1–28 days.

Subsequently, we randomly assigned the 80 samples in each square to five groups of size 16, thus creating training sets with 64 samples in each square and test sets with 16 samples in each square. We applied the SEDLM algorithm to all five train- and test set combinations. To avoid artefacts at the edge of *S*, we calculated the posterior probabilities only for points lying within the 30$\,\times \,$30 inner squares.

Finally, to examine the effect of the envelope, we created an additional set of simulations on a rectangle with entry effect range from −0.04 to 0.04 and exit effect from −0.02 to 0.02. We used the same size for the inner grid cells and generated 64 time series per cell. These were used to re-estimate the smooth functions with the larger envelope and provide new SEDLM estimates for the original 81,920 test samples.

## Results

**Table 1:** Simulation results: RMSE $\times$ 500 for estimated versus simulated entry and exit effects for various (SE)DLM specifications: the maximum lag *L* included and restrictions upon the lag structure

Maximum lag	10	20	10 p4	20 p4	40 p4	60 p4	10 bs5	20 bs5	40 bs5	60 bs5	60 bs10
*Entry effect*											
DLM	4.11	2.79	4.15	2.59	2.85	5.01	4.14	2.55	3.07	5.27	1.91
SEDLM	1.62	1.20	1.61	1.13	0.98	1.00	1.61	1.13	0.99	1.03	0.93
*Exit effect*											
SEDLM	0.34	0.33	0.35	0.36	0.63	0.92	0.35	0.38	0.70	0.97	0.36
*Combined*											
SEDLM	1.65	1.24	1.65	1.19	1.16	1.36	1.65	1.19	1.21	1.41	1.00

p4: polynomial of 4th degree. bs5 and bs10: cubic B-spline with five degrees of freedom, respectively ten. For compatibility between DLM and SEDLM, only points lying in the 30 × 30 inner squares were used

Table 1 shows the results of the simulation. We tested DLMs with multiple choices of *L* and both with and without restrictions. As the number of lag terms increases, the root mean squared error (RMSE) of the unconstrained DLMs decreases. Thus the DLMs’ ability to capture the full effect of exposure increases with the number of lag terms included. The performance of restricted DLMs is similar to the unconstrained DLMs with equal amount of lag terms, however the difference is rather small. For larger amounts of lagged terms, the entry performance decreases again unless the restrictions are lessened.

**Fig. 5:**
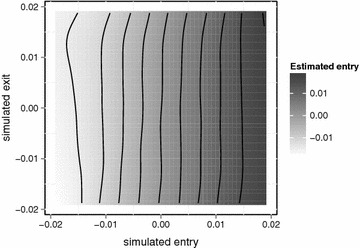
Heatmap of entry-estimates in function of simulated entry and exit effects for a SEDLM with $L=40$ and B-spline restriction on the lag coefficients ($df=5$)

Comparing SEDLM with DLM, all SEDLMs have much lower RMSE than the corresponding DLM. SEDLMs based on restricted DLMs perform slightly better than SEDLMs based on unconstrained DLMs. The best SEDLM has a RMSE that is two times smaller than that of the best DLM. Furthermore, the difference in RMSE between the SEDLMs is much smaller than the difference between the DLMs. Comparison of Figs.  4 and 5 confirms that SEDLM entry estimates are a function of simulated entry that is less perturbed by simulated exit.

SEDLMs provide information both on the entry and the exit effect. Results in Table 1 indicate that the algorithm’s ability to capture the exit effect is even better than the accuracy of the entry effect estimates.

**Fig. 6:**
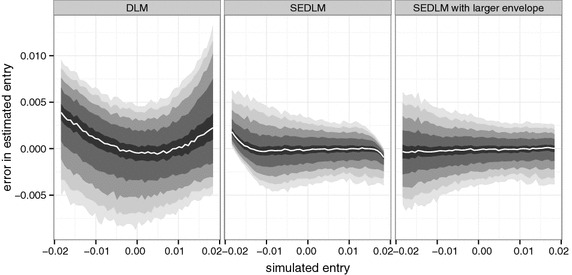
Binned quantile plot of error in estimated entry effect. *Left*: DLM with lags 0–60, restricted to a B-spline basis with 10 degrees of freedom. *Middle* corresponding SEDLM with square envelope. *Right* corresponding SEDLM with larger, rectangular envelope. The bands contain respectively 95, 90, 80, 60 and 20%; the *white line* represents the median

**Fig. 7:**
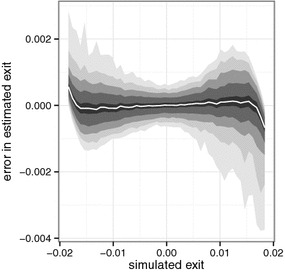
Binned quantile plot of error in estimated exit effect. SEDLM with lags 0–60, restricted to a B-spline basis with 10 degrees of freedom. The bands contain respectively 95, 90, 80, 60 and 20%; the *white line* represents the median

Figure 6 provides further insight into the advantages of SEDLMs. Clearly the DLM has a larger variance than the SEDLM. Both methods have negligible bias near the center, however this particular DLM suffers from a non-linear attenuation effect for negative entry, whereas positive entry is overestimated. For the SEDLM the bias remains negligible for a larger range. However near to the edges of the envelope attenuation occurs. This effect can be mitigated by using a larger envelope at the cost of increased computational burden and a small variance penalty as is visible in the right panel.

Figure 7 reveals a similar pattern for the exit effect. The exit effect is estimated without bias over most of the simulation range, yet some bias remains near to the edges of the envelope. The pattern is similar to that of the entry effect, but the variance is much lower than that of the entry effect and the influence of the envelope is less extended.

**Fig. 8:**
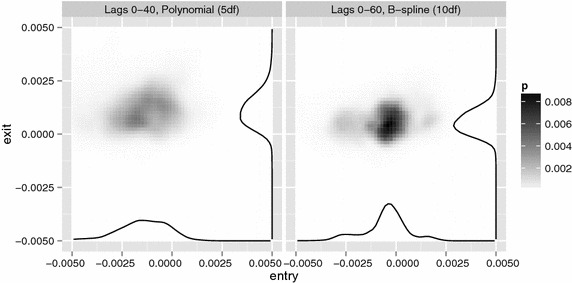
Posterior probability for Chicago, calculated with two SEDLMs. *Left* base model includes lags 0 up until 40, restricted to a 4th degree polynomial. *Right* base model includes lags 0 up until 60, restricted to a cubic B-spline with 10 degrees of freedom

We analysed the data of Chicago with the two best performing SEDLMs. Figure 8 shows the posterior probability density functions as well as the marginals. Zero lies within each marginal highest posterior density interval; these results do not provide evidence of an entry or an exit effect.

Both specifications provide similar posteriors. Furthermore, the results are consistent with Table 1: the accuracy of the exit effect estimate is higher than that of the entry effect estimate, especially for the model with lags 0–60. Similar results were obtained using SEDLM with other lag specifications. To further test sensitivity, we repeated the procedure using subsets of the training set and using a secondary training set wherein the mean life time in the frail state was changed to 1 + Poisson(14). No meaningful changes were observed.

## Discussion

The SEDLM algorithm developed in this paper has multiple benefits over DLMs. Primarily, it provides unbiased estimates. Secondly, it reduces the variance of the estimated entry effect. It also gives an additional estimate of the exit effect. Finally, it illustrates the issue of the strong assumptions necessary to use the sum of lag estimates as an estimate of the entry effect. However, besides these assumptions, other user inputs are required both for DLM and SEDLM.

First, DLMs can be sensitive to the number of lag terms *L* included in the model. The choice of *L* is based on user experience, previous results and intuition about the mean life time in the frail population. Similarly, prior information about the MLT is required for SEDLM. Although our results were not sensitive to changing the mean of the distribution, there may exist sensitivity to more general changes or for other datasets. Even though the SEDLM approach might be generalized to derive a posterior for the MLT too, a prior distribution for the MLT will still need to be chosen. Using at least three non-parallel surfaces $f_j^{\mathrm {DLM}} \left( \beta _{\mathrm {entry}},\beta _{\mathrm {exit}}, {\mathrm {MLT}}\right)$, such a generalization is conceptually straightforward, but the required three dimensional grid will impose a much larger computational challenge than the two dimensional grid we used. As grid based approaches do not scale well with dimensionality, such extensions are probably impractical.

A second necessary choice for DLMs is the number of degrees of freedom for the seasonal effects. This can be made a priori in GLMs or by optimizing a selection criterion when GAMs are used. For SEDLM, this choice is moved to the Monte Carlo part of the three step process, wherein all time series were generated using the same seasonal smooth. Again, it is straightforward to extend the algorithm to include multiple seasonal functions.

For SEDLM only, an envelope needs to be chosen. It is clear that SEDLM can produce unbiased estimates only when the true effects lie within the envelope. The simulation results show how far from the edge these true effects ought to be. Thus SEDLM can always be made unbiased by increasing the envelope’s size, at the cost of extra computations. Currently, fitting a DLM to each time series, is the most time-consuming part. Fortunately, this step is ‘embarrassingly parallel’. Using eight threads allowed us to fit a set of 81,920 time series in less than three hours. The total computational cost increases slightly when unconstrained DLMs are used. The other steps, simulating the data, smoothing the response surfaces and performing the numerical integration, all take mere minutes or seconds with our code. Thus, in our implementation, the total computational burden depends linearly on the number of simulations; in essence on the size of the envelope.

The envelope can also be regarded as prior information on the entry and exit effects. They are uniform within the envelope and zero outside. Any bounded prior can be used with our algorithm and using a more informative prior may be advantageous when combining results from multiple populations and cities. Because the changes occur only in the final step, they can be applied without intensive computations, provided that the boundaries themselves are not changed.

Another difference with DLMs is the necessity to specify a three state model. The model used in this work is the most simple non-degenerate three state model. More complex models can allow for bypassing the frail state, the existence of multiple frail states (Roberts 2011) and non-linear entry and exit effects (Roberts 2011). In addition, the entry and exit effects themselves are sometimes treated as distributed lags. Although such does not strike us as parsimonious, the other variations cannot be as easily dismissed and must therefore be investigated. While not explicitly tested, it is unlikely that SEDLM estimates obtained from a poorly-specified three state model will be accurate and unbiased. DLMs however, remain agnostic with respect to the multi state model, even though they are sensitive to the underlying data generating process. Some processes, including two state models, will not result in biased DLM estimates. Others can result in attenuation or overestimation.

Although SEDLM does not yield perfect answers for all choices a DLM necessitates, it provides better estimates without large downsides. These estimates have consistent frequentist properties both with respect to bias and standard deviation. Noting that DLMs are the most frequently used models, the case for SEDLM is favorable. Other alternatives for DLM have been discussed by Murray and Lipfert (2010). They partition the methods into zero-sum studies and compartment models.

DLMs are zero-sum studies, as are Frequency Domain LogLinear regression (Kelsall et al. 1999) and Timescale LogLinear regression (Dominici et al. 2003). Fung et al. (2005) investigated the robustness of the latter method to data generated by a three state model and concluded that ‘time scale regression has limited value for detecting mortality displacement in time-series data.’ No results are available for Frequency Domain LogLinear regression, but the method is quite similar to the other zero-sum approaches and we suspect it performs similarly.

Besides SEDLM, two other compartment models exist. The Kalman filter is relatively unknown to air pollution epidemiologists, having its origins in electronics. It can be applied to derive estimates of the exit effect and the mean lifetime in the frail population (Murray and Lipfert 2010; Murray and Nelson 2000). However, the few documented implementations that we are aware of, used the assumption that the entry effect is null. This makes the Kalman filter a good candidate for combination with SEDLM. The former provides estimates of the MLT, which can be used as input for the latter to derive joint estimates of the entry and exit effects, assuming one is interested in the entry effect.

Smith et al. (1999) used a full Bayesian approach to derive simultaneous estimates of the MLT, entry and exit effects. Because of the computational intensity of the Markov Chain Monte Carlo, they were unable to check convergence in their simulation study. One iteration failed to converge altogether, rendering interpretation of the results quite difficult: the posterior standard deviations are too small when compared with the frequentist properties.

When applying the SEDLM to Chicago data, the highest posterior density intervals for entry and exit effects both included zero. In other words, there is no evidence for a ‘net’ effect—an effect of air pollution on the general population of residents of age 65 and above—and, neither is there evidence for a displacement effect. From the non-significant entry estimate it follows that the impact on life expectancy at birth is not significant. Even though the exit effect estimate is not significant, however, it cannot be concluded that the three state model must be degenerate. Because the exit effect represents the change in exit probability due to air pollution and not the base probability of exit, it remains possible that the latter is not equal to one. Further investigation of this would require a combination with other approaches such as the Kalman filter, extension of the SEDLM method or development of a full Bayesian method. In addition to this and the issues outlined above, the interpretation of the SEDLM estimates keeps the traditional caveats: non/significant effects may be due to lack of power or bias through misspecification of seasonal components or confounders.

## Conclusion

SEDLM can be considered a compartment model whose posterior estimates have consistent frequentist properties. The results of our simulation study allow us to be optimistic about the algorithm as well as the other compartment based approaches.

SEDLM was developed when investigating the origin of the bias that DLMs suffer. By modifying one assumption, the SEDLM significantly improves upon the DLM in terms of mean squared error. This boon is the sum of a reduction in bias and a reduction in standard deviation. In addition to more accurate estimates of the entry effect, SEDLM also delivers simultaneous estimation of the exit effect. The exit estimate is even more accurate than the entry effect estimate. This provides valuable quantitative information on the mortality displacement hypothesis.

Besides a compartment based, full Bayesian approach, no other method for simultaneous estimation is available and SEDLM is currently the only approach that is feasible. These results warrant further investigation into SEDLM and/or the full Bayesian approach.

## Additional file

The reader is referred to the on-line Additional file for R code (Additional file 1).

## Additional file

**Additional file 1.** Code used for the simulations and creation of tables and figures in the article.
